# Chromosome-level genome assembly of the medicinal insect *Blaps rhynchopetera* using Nanopore and Hi-C technologies

**DOI:** 10.1093/dnares/dsae027

**Published:** 2024-09-09

**Authors:** Wei Zhang, Yue Li, Qi Wang, Qun Yu, Yuchen Ma, Lei Huang, Chenggui Zhang, Zizhong Yang, Jiapeng Wang, Huai Xiao

**Affiliations:** Yunnan Provincial Key Laboratory of Entomological Biopharmaceutical R&D, Dali 671000, Yunnan Province, People’s Republic of China; National-Local Joint Engineering Research Center of Entomoceutics, Dali 671000, Yunnan Province, People’s Republic of China; College of Pharmacy, Dali University, Dali 671000, Yunnan Province, People’s Republic of China; Yunnan Provincial Key Laboratory of Entomological Biopharmaceutical R&D, Dali 671000, Yunnan Province, People’s Republic of China; National-Local Joint Engineering Research Center of Entomoceutics, Dali 671000, Yunnan Province, People’s Republic of China; College of Pharmacy, Dali University, Dali 671000, Yunnan Province, People’s Republic of China; School of Artificial Intelligence and Information Technology, Nanjing University of Chinese Medicine, Nanjing 210023, Jiangsu Province, People’s Republic of China; Yunnan Provincial Key Laboratory of Entomological Biopharmaceutical R&D, Dali 671000, Yunnan Province, People’s Republic of China; National-Local Joint Engineering Research Center of Entomoceutics, Dali 671000, Yunnan Province, People’s Republic of China; College of Pharmacy, Dali University, Dali 671000, Yunnan Province, People’s Republic of China; Yunnan Provincial Key Laboratory of Entomological Biopharmaceutical R&D, Dali 671000, Yunnan Province, People’s Republic of China; National-Local Joint Engineering Research Center of Entomoceutics, Dali 671000, Yunnan Province, People’s Republic of China; College of Pharmacy, Dali University, Dali 671000, Yunnan Province, People’s Republic of China; Yunnan Provincial Key Laboratory of Entomological Biopharmaceutical R&D, Dali 671000, Yunnan Province, People’s Republic of China; National-Local Joint Engineering Research Center of Entomoceutics, Dali 671000, Yunnan Province, People’s Republic of China; College of Pharmacy, Dali University, Dali 671000, Yunnan Province, People’s Republic of China; Yunnan Provincial Key Laboratory of Entomological Biopharmaceutical R&D, Dali 671000, Yunnan Province, People’s Republic of China; National-Local Joint Engineering Research Center of Entomoceutics, Dali 671000, Yunnan Province, People’s Republic of China; College of Pharmacy, Dali University, Dali 671000, Yunnan Province, People’s Republic of China; Yunnan Provincial Key Laboratory of Entomological Biopharmaceutical R&D, Dali 671000, Yunnan Province, People’s Republic of China; National-Local Joint Engineering Research Center of Entomoceutics, Dali 671000, Yunnan Province, People’s Republic of China; College of Pharmacy, Dali University, Dali 671000, Yunnan Province, People’s Republic of China; Yunnan Provincial Key Laboratory of Entomological Biopharmaceutical R&D, Dali 671000, Yunnan Province, People’s Republic of China; National-Local Joint Engineering Research Center of Entomoceutics, Dali 671000, Yunnan Province, People’s Republic of China; College of Pharmacy, Dali University, Dali 671000, Yunnan Province, People’s Republic of China; Yunnan Provincial Key Laboratory of Entomological Biopharmaceutical R&D, Dali 671000, Yunnan Province, People’s Republic of China; National-Local Joint Engineering Research Center of Entomoceutics, Dali 671000, Yunnan Province, People’s Republic of China; College of Pharmacy, Dali University, Dali 671000, Yunnan Province, People’s Republic of China; Yunnan Provincial Key Laboratory of Entomological Biopharmaceutical R&D, Dali 671000, Yunnan Province, People’s Republic of China; National-Local Joint Engineering Research Center of Entomoceutics, Dali 671000, Yunnan Province, People’s Republic of China; College of Pharmacy, Dali University, Dali 671000, Yunnan Province, People’s Republic of China

**Keywords:** chromosome-level genome assembly, *B. rhynchopetera*, Hi-C, Nanopore

## Abstract

*Blaps rhynchopetera* Fairmaire is a significant medicinal resource in southwestern China. We utilized Nanopore and Hi-C technologies in combination to generate a high-quality, chromosome-level assembly of the *B. rhynchopetera* genome and described its genetic features. Genome surveys revealed that *B. rhynchopetera* is a highly heterozygous species. The assembled genome was 379.24 Mb in size, of which 96.03% was assigned to 20 pseudochromosomes. A total of 212.93 Mb of repeat sequences were annotated, and 26,824 protein-coding genes and 837 noncoding RNAs were identified. Phylogenetic analysis indicated the divergence of the ancestors of *B. rhynchopetera* and its closely related species *Tenebrio molitor* at about 85.6 million years ago. The colinearity analysis showed that some chromosomes of *B. rhynchopetera* may have had fission events, and it has a good synteny relationship with *Tribolium castaneum*. Furthermore, in the enrichment analyses, the gene families related to detoxification and immunity of *B. rhynchopetera* facilitated the understanding of its environmental adaptations, which will serve as a valuable research resource for pest control strategies and conservation efforts of beneficial insects. This high-quality reference genome will also contribute to the conservation of insect species diversity and genetic resources.

## 1. Introduction

*Blaps rhynchopetera* Fairmaire (Coleoptera: Tenebrionidae) is a medicinal insect in southwest China. Although *B. rhynchopetera* has no flight function, it can secrete defensive foul-smelling liquid in emergency situations, so it is commonly called the ‘smelly fart bug’ or ‘stink beetle’.^1^ This insect is abundantly found in Yunnan Province, particularly concentrated in the central and eastern areas. *B. rhynchopetera* has long been used for tumours, gastritis, fever, and cough by native Bai and Yi nationalities of Yunnan of China.^2–4^ In recent years, the chemical composition and medicinal value of *B. rhynchopetera* have been increasingly researched and explored. Extracts from this beetle have exhibited immunomodulatory,^5^ treatment for vaginal inflammation,^6^ and antibacterial effects.^7^ Moreover, the defensive secretion of *B. rhynchopetera* may be an important source of tumour drug development and biomedicine.^8^ In particular, quinones that were found in defence secretion had significant proliferation inhibitory and proapoptotic effects on colorectal tumour cells.^9^ Professional artificial breeding has been conducted, altering the previous reliance on wild collection and expanding the potential utilization of this insect to meet the medicinal needs of people.

With the rapid development of sequencing technologies, the genomic information of an increasing number of species has been acquired and analysed. Deep sequencing data provide extensive information about the genome and gene expression profiles, enabling researchers to explore the processes of life from both the global and local perspectives. Currently, there are some medicinal insect species with completed genome sequencing results published or deposited in the public database National Center for Biotechnology Information (NCBI). These species span across nine orders, including Blattodea, Orthoptera, Hemiptera, Coleoptera, and Lepidoptera, among others (NCBI, accessed 9 March 2024).^10^ High-quality genome can not only provide new insights into evolutionary characteristics, phylogeny, and environmental adaptability of medicinal insects but also hold crucial significance for the preservation of insect species diversity and genetic resources. However, only transcriptome and mitochondrial genomes have been assembled in *B. rhynchopetera* to date (NCBI, accessed 4 June 2024).^11,12^ Further research on this species is hindered due to the lack of a high-quality genome.

Here, we generated a high-quality genome assembly of *B. rhynchopetera* by combining third-generation sequencing (Nanopore sequencing) technologies^13^ and Hi-C technology,^14^ complementing the chromosomal-level genomic information of Coleoptera. The acquisition and utilization of a range of genetic resources from *B. rhynchopetera* will contribute to a better understanding of its evolutionary history, environmental adaptation, and the habits, helping farmers and businesses in formulating breeding strategies. This study also provides important genomic resources for future population genetics, adaptive evolution, genetic differentiation, genetic manipulation, and mining of medicinal genes of *B. rhynchopetera*.

## 2. Materials and methods

### 2.1. Samples and sequencing

During this study, adult samples of *B. rhynchopetera* were carefully selected from the farm market of Dali in Yunnan Province, China, and were identified by Professor Zizhong Yang. To minimize the risk of contamination, the genomic DNA was extracted from the muscles of the thoracic or foot regions using the sodium dodecyl sulfate (SDS) lysis buffer extraction technique, purified using Ampure XP beads and purification columns (OMEGA). Additionally, the RNA was extracted following the standard operating procedures outlined by the TRIzol reagent.

For next-generation sequencing (NGS) (short reads), a short-insert (150-bp) genomic library was performed using the Nextera DNA Flex Library Preparation Kit (Illumina, San Diego, CA, USA) and sequenced on the MGI DNBSEQ-T7 platform using short-reads paired-end sequencing strategy. The raw reads obtained from sequencing were filtered utilizing the Fastp (v.0.21.0)^15^ to obtain clean reads. The reads filtering criteria are as follows: (i) with N bases more than 5%, (ii) having more than 50% bases with Phred quality < 5, (iii) with adapters, and (iv) PCR-duplicated reads.

For long-reads Nanopore sequencing, 2 distinct libraries were prepared, genomic DNA and cDNA of RNA transcription. Subsequently, the genomic and transcriptomic libraries underwent single-molecule real-time sequencing on an Oxford Nanopore PromethION P48 sequencer. The raw reads generated from sequencing were filtered by removing sequences with the mean_qscore_template≤7 to obtain clean reads.

The high-throughput/resolution chromosome conformation capture (Hi-C) sequencing is utilized to explore the spatial relationships of the entire chromosomal DNA within the chromatin.^14^ The Hi-C library was sequenced on the Illumina NovaSeq 6000 platform with the layout of paired-end 150-bp reads, and the filtering criteria were the same as NGS (short reads). Clean data from Hi-C sequencing were aligned with draft genome. HiCUP (v.0.8.0)^16^ was utilized to remove unmapped reads, which exhibited nonunique alignment at both ends of the reference genome. In addition, it removed invalid pairs, self-circular sequences, dangling ends, and PCR amplification-derived repeats. All library construction and sequencing were conducted in Benagen Tech Solutions Company Limited (Wuhan, China).

### 2.2. Genome survey and de novo assembly

An analysis with *k*-mer = 19^17^ was conducted on all clean reads obtained from NGS (short reads) sequencing to estimate the genome size and heterozygosity of *B. rhynchopetera* using GCE (v.1.0.0)^17^ and Jellyfish (v.2.2.10).^18^ To assess the potential contamination of the sequencing data, a subset of 50,000 reads was compared against the NCBI NT database (v.202107) using Blast (v.2.11.0+) (https://blast.ncbi.nlmr.nih.gov/Blast.cgi).

The clean reads from Nanopore sequencing were assembled using NextDenovo.^19^ The primary genome was corrected by Racon (v.1.4.1) (https://github.com/isovic/racon) and Pilon (v.1.23).^20^ To get the genome assembly of the highly heterozygous species, the corrected genome was de-heterozygote using Purge haplotigs (v.1.0.4).^21^ BUSCO (v.4.1.2) (insecta_odb10, fly)^22^ was used to assess the genomic completeness.

For chromosome assembly, ALLHiC^23^ was used to cluster the contig sequences into different chromosome groups using agglomerative hierarchical clustering. The contigs within each chromosome group underwent ordering and orientation. 3D-DNA (v.180419)^24^ and Jucier (v.1.6)^25^ convert the pairwise interactions among contigs into a Hi-C file, and Juicebox^26^ was utilized to manually order and orient the contigs. After the removal of heterozygous sequences, the final chromosome-level genome sequence was obtained by filling the gaps with 100 N. Finally, HiCExplorer (v.3.6)^27^ was used to visualize the strength and positional relationships among the contigs.

### 2.3. Genome annotation

RepeatModeler open-1.0.11^28^ was used to predict the model sequence, and LTR_FINDER^29^ predicted long terminal repeat (LTR) sequences. After that, we constructed a de novo repeat library according to the results predicted by 2 pieces of software. Based on the results of merging the Repbase library (v.20181026)^30^ with the de novo library, we utilized RepeatMasker open-4.0.9^31^ to identify repeat sequences. In addition, RepeatProteinMask open-4.0^31^ was employed to predict TE (transposable element) protein-type repeat sequences. In conclusion, we amalgamated all predicted results to obtain the definitive genomic repeat sequence.

We employed a combination of transcriptome prediction, homology prediction, and de novo prediction to predict the protein-coding genes of *B. rhynchopetera*. Transcriptome prediction was achieved through third-generation transcriptomes (Nanopore long reads). Based on the clean reads generated by Nanopore RNA sequencing, the genome was aligned using minimap2 (v.2.17-r941).^32^ The resulting alignments were then converted into BAM files. Transcriptome assembly was performed using StringTie (v.2.1.4),^33^ and the predicted transcript regions were further analysed to predict coding frames using TransDecoder (v.5.1.0), ultimately yielding the predicted coding genes. Homology prediction was based on protein sequence files from *Tenebrio molitor* and *T. castaneum*. According to a genome masked by repetitive sequences, de novo prediction was conducted using Augustus (v.3.3.2). MAKER (v.2.31.10)^34^ integrated the gene sets obtained from the 3 prediction methods, verifying the accuracy of predicted open reading frames, the starting and ending positions of coding regions, and gene lengths. Finally, we used a comprehensive database to predict the biological functions and acquire metabolic pathway information for the sequences of protein-coding genes, specifically nonredundant,^35^ UniProt,^36^ gene ontology (GO),^37^ Kyoto Encyclopedia of Genes and Genomes (KEGG),^38^ Pfam,^39^ and InterProScan.^40^

The ribosomal RNAs (rRNAs) were predicted using the rRNA database, while tRNAscan-SE (v.1.23)^41^ was utilized to find transfer RNAs (tRNAs) sequences in the genome. Small nuclear RNAs (snRNAs) and microRNAs (miRNAs) sequences were annotated using INFERNAL (v.1.1.2)^42^ according to the Rfam database.^43^ In addition, the completeness of genome annotation was assessed using BUSCO (v.5.2.2) (lineage: endopterygota_odb10).^22^

### 2.4. Genome evolution analysis

Orthologous genes among *B. rhynchopetera* and 11 other species were identified using OrthoFinder.^44^ After collating information on gene families, MUSCLE (v3.8.31)^45^ was utilized to align the protein sequences of each single-copy gene family. The unique gene families were subjected to GO and KEGG enrichment analysis using clusterProfiler57 (v.3.6.0).^46^ In addition, to illustrate the phylogenetic relationships among 12 insect species, protein sequences of single-copy gene families were aligned, with the number of single-copy genes set at 913. A species phylogeny tree was constructed using RAxML (v.8.2.10).^47^ Subsequently, divergence times of the species were estimated using the MCMCtree subroutine of the PAML package,^48^ with parameters set as follows: nsample = 3,000,000; burnin = 8,000,000; seqtype = 0; model = 4. Time points for calibration were obtained from the TimeTree database (http://www.timetree.org/), primarily focussing on the following intervals: *Tribolium madens* versus *Coccinella septempunctata* (166.9–188.2 million years ago [Mya]), *Photinus pyralis* versus *C. septempunctata* (204.4–237.0 Mya), *Diabrotica virgifera* versus *Anthonomus grandis* (129.6–245.8 Mya), and *Drosophila melanogaster* versus *Bombyx mori* (223.8–344.7 Mya).

We employed Computational Analysis of gene Family Evolution (CAFE, v.3.1)^49^ to analyse the expansion and contraction of gene families within *B. rhynchopetera*. In CAFE, the birth–death rate model was used to estimate the quantity of gene family members in each ancestral branch, thereby predicting the expansion and contraction of gene families in relation to the ancestors of *B. rhynchopetera*. A threshold of *P* value < 0.05 was established as the criteria for identifying significant expansions or contractions. In addition, an enrichment analysis of KEGG pathways was performed on the expanded gene families within *B. rhynchopetera*.

### 2.5. Synteny analysis

We utilized the alignment software LAST (v.1170)^50^ to identify gene pairs exhibiting similarity across species. Subsequently, JCVI (v.0.9.13)^51^ was utilized to determine whether the similar gene pairs were adjacent on the chromosome, leveraging the annotation file (gff3) as a reference. The findings were then visualized to offer a comprehensive understanding of the genetic relationships.

### 2.6. Positive selection

Utilizing the software MAFFT,^52^ the protein sequences of the identified single-copy gene families among the chosen species were aligned. Subsequently, PAL2NAL (v.14)^53^ was used to convert the protein sequences into codon alignments. The CodeML program, incorporated within PAML (v4.9),^48^ was executed using the branch-site model to determine whether particular genes were subjected to positive selection in *B. rhynchopetera*. Genes exhibiting *P* value < 0.05 were considered positive selection genes (PSGs) in *B. rhynchopetera*.

## 3. Results and discussion

### 3.1. Genome sequencing and survey

Before Nanopore sequencing, NGS (short reads) generated about 104.03 clean bases, which were used for sample quality and genome assessment. Utilizing the depth frequency distribution analysis of k-mer = 19 (Supplementary Fig. S1), it was estimated that the genome size of *B. rhynchopetera* approximates 431 Mb, with a heterozygosity ratio of 1.94% and a repetition ratio (Supplementary Table S1). Due to these characteristics, *B. rhynchopetera* is recognized as a species exhibiting a high degree of heterozygosity. In addition, the comparison with the NT database revealed the absence of notable external contamination in the samples (Supplementary Table S2). Consequently, the samples from this research are deemed suitable for Nanopore sequencing and genome assembly. Through the utilization of Nanopore sequencing and Hi-C sequencing, respectively, clean bases totalling 48.38 Gb and 59.41 Gb were obtained (Table 1).

**Table 1.** Statistics regarding the genome and RNA sequencing data of *B. rhynchopetera* (bp).

Category	Total reads	Raw bases	Clean bases	N50 reads length
NGS (short reads)	713,151,418	106,972,903,200	104,034,608,390	–
Nanopore	4,907,734	57,334,027,437	48,380,116,953	35,674
Hi-C	399,331,678	59,987,419,500	59,413,053,714	–
Nanopore RNA	12,224,672	11,963,799,551	11,099,847,055	–

### 3.2. Genome assembly

Based on the Nanopore sequencing data, the genome of *B. rhynchopetera* was preliminarily assembled through the NextDenovo software. The manually corrected genome assembly resulted in 136 contigs with a total length of 379.24 Mb and a contig N50 of 12.02 Mb (Table 2). To validate the quality of the assembly, we aligned the NGS (short reads) data with the assembly, obtaining an alignment rate of 99.92% and a coverage rate of 99.81%. In addition, through the BUSCO assessment (*n* = 1,367), we identified 1,355 complete genes (99.1%) in the assembly, with 97.4% being ‘Complete and single-copy BUSCOs’ and 1.7% being ‘Complete and duplicated BUSCOs’ (Table 2). These results indicate a remarkable degree of consistency and completeness in the genome assembly of *B. rhynchopetera*.

**Table 2.** Statistics regarding the genome assembly and annotation of *B. rhynchopetera*.

Parameter	Number
**Genome assembly**	
Assembly size (bp)	379,239,822
Number of scaffolds/contigs	79/136
N50 scaffold/contig length (Mb)	22.03/12.02
Longest scaffold/contig (Mb)	26.49/22.57
Average scaffold/contig length (bp)	4,800,504.08/2,971,644.51
GC (%)	35.34
BUSCO completeness (%)	99.1
**Gene annotation**	
Repeat sequences length (Mb)	212.93
Protein-coding genes	26,824
Total number of exon/intron	90,773/63,949
Exons per gene	3.38
The average length of mRNA/CDS (bp)	6,466.54/901.73
Number of miRNAs/tRNAs/rRNAs/snRNAs	51/404/292/90
BUSCO completeness (%)	94.0

**Fig. 1.**
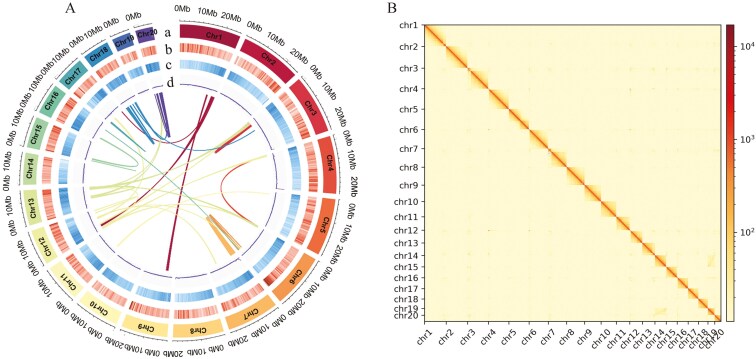
Genome assembly of *B. rhynchopetera* genome. A) Circos graph of *B. rhynchopetera* genetic features (window size = 50 kb), showing (a) length of assembled chromosomes, (b) gene density, (c) repeat sequence density, and (d) GC content. Lines in the centre of the circle indicate syntenic blocks. B) Hi-C heatmap of *B. rhynchopetera* chromosome interactions.

To achieve a chromosome-level assembly, based on the clean reads generated by HI-C sequencing, the contigs were anchored to the pseudochromosome using the Hi-C scaffolding approach. A sequence totalling 364,162,369 bp was located onto 20 pseudochromosomes, accounting for 96.03% of the entire assembly (Fig. 1A) (Supplementary Table S3). The corresponding heatmap analysis revealed a clear distinction among the 20 chromosome groups, characterized by intense interactions within each chromosome. Notably, no abnormal interaction signals were detected, thereby affirming the high quality of our Hi-C-assisted genome assembly (Fig. 1B). The chromosome-level assembly of *B. rhynchopetera* genome yielded 79 scaffolds, with an N50 length of 22.03 Mb and an average scaffold length of 4,800,504.08 bp (Table 2). These statistical figures verified the precision, completeness, and overall superior quality of our genome assembly at the chromosome level. The final genome sequence has been submitted to GenBank under accession number JAYJMQ00000000.

### 3.3. Gene annotation

A total of 212.93 Mb of repeat sequences were annotated, accounting for 56.15% of the entire genome (Fig. 1A). Unclassified elements (32.50%), DNA elements (10.78%), long interspersed nuclear element (LINE, 8.74%), and LTR (2.98%) were the 4 repeat sequence classes with the highest abundance (Supplementary Table S4). The high proportion of unclassified elements is attributed to the lack of research on repetitive sequences in *B. rhynchopetera*.

The genetic structure of *B. rhynchopetera* was predicted through a combination of transcriptome prediction, homology prediction, and de novo prediction, resulting in 26,824 protein-coding genes, 90,773 exons, and 63,949 introns, respectively. The average length of the mRNA and CDS (coding sequence) was 6,466.54 bp and 901.73 bp, respectively (Table 2). An analysis comparing the length distribution of genes and CDS introns and exons in the *B. rhynchopetera* genome with those of closely related species has been conducted. The results indicate a comparable frequency distribution of gene and intron length across species. However, some notable differences were observed in the gene length distribution when compared with the *T. molitor* genome (Supplementary Fig. S2). Multiple databases have provided annotations for the protein-coding genes of *B. rhynchopetera*. Specifically, the Nr, KEGG, GO, UniProt, KEGG Pathway, Pfam, and InterProScan databases have collectively annotated 19,078 protein-coding genes, with 2,961 genes receiving consistent annotations across these databases. Precisely, there is a correspondence between 15,746 annotated genes and one or more records in the UniProt database. Furthermore, 4,371 genes have been annotated in the KEGG, 4,371 in Nr, 11,432 in GO, 3,381 in KEGG Pathway, 10,699 in Pfam, and 16,933 in the InterPro public database (Supplementary Table S5). In addition to protein-coding genes, our analysis revealed the presence of 837 noncoding RNAs (ncRNAs), encompassing 51 microRNAs (miRNAs), 404 tRNAs, 292 ribosomal RNAs (rRNAs; including 18S, 28S, 5.8S, and 5S), and 90 small nuclear RNAs (snRNAs). Furthermore, we identified 63 RNA splicing factors, 22 CD-box small nucleolar RNAs (snoRNAs), 4 HACA-box snoRNAs, and 1 small Cajal body-associated RNA (scaRNA) within the snRNA group (Supplementary Table S6). In the BUSCO assessment, the annotation completeness of genes attained a level of 94.0%, with 93.2% being ‘Complete single-copy BUSCOs’ and 0.8% being ‘Complete duplicated BUSCOs’.

### 3.4. Comparative genomic analysis and phylogeny

**Fig. 2.**
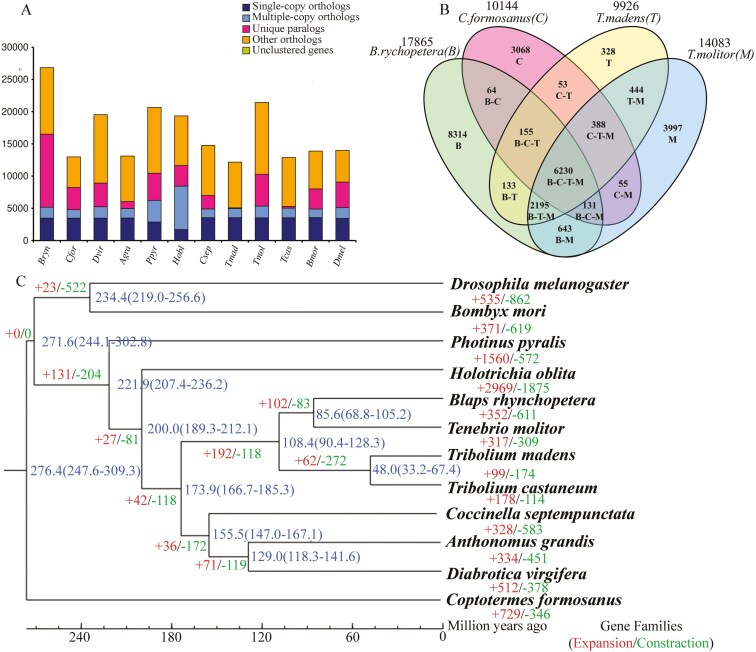
Comparative genomic analysis and phylogeny. A) Statistical results on the number of homologous genes across species. This includes the number of single-copy homologous genes within gene families shared by species (designated as single-copy orthologs), the number of multicopy homologous genes within these shared gene families (multicopy orthologs), genes that do not cluster into any family (unclustered genes), genes belonging to species-specific gene families (unique paralogs), and all other genes (other orthologs). B) Statistical Wayne plots of gene family clustering results across 4 species. Values indicate the number of gene families present; the letters in parentheses represent the abbreviations of the Latin name of the species; the strings of abbreviated links indicate gene families that are commonly shared among these species, while the single abbreviated letters indicate the number of gene families unique to each species within the given 4 species. C) Phylogenetic tree. An analysis of the phylogenetic tree and divergence times of *B. rhynchopetera*, based on 913 single-copy orthologs, reveals that the length of each branch represents the duration of time elapsed and corresponds directly to the timeline.

In the pursuit of identifying homologous genes, conducting gene family clustering analysis, and examining the enrichment of single-copy and multicopy genes, a diverse selection of species was made. This included *B. rhynchopetera*, 8 Coleopteran species (*Tmol*, *T. molitor*; *Tmad*, *Tribolium madens*; *Tcas*, *Tribolium castaneum*; *Agra*, *A. grandis*; *Dvir*, *D. virgifera*; *Csep*, *C. septempunctata*; *Hobl*, *Holotrichia oblita*; *Ppyr*, *P. pyralis*) and 3 other species (*Dmel*, *D. melanogaster*; *Bmor*, *B. mori*; *Cfor*, *Coptotermes formosanus*) (Fig. 2A). The comprehensive analysis revealed the existence of 38,333 orthologous gene families across all species, encompassing a total of 201,515 genes, including 913 single-copy gene families. Furthermore, 3,986 gene families were shared among all species, representing 65,093 genes. The genome of *B. rhynchopetera* contained 17,865 gene families, some of which may also exist in other insects. Notably, it possessed 8,040 unique gene families, comprising 11,333 genes (Supplementary Table S7). A comparative analysis of gene families was conducted among 2 closely related species, *T. molitor* and *T. madens*, as well as an outgroup species, *C. formosanus* (Fig. 2B). The results indicated a total of 6,320 gene families shared among these species. Specifically, *B. rhynchopetera* exhibited 9,199 and 8,713 gene families in common with *T. molitor* and *T. madens*, respectively. However, its similarity with *C. formosanus* was slightly lower, as they shared 6,580 gene families. Notably, *B. rhynchopetera* possessed 8,314 unique gene families.

A phylogenetic tree was constructed based on 913 single-copy orthologs utilizing RAxML (v.8.2.10). As shown in Figure 2B, the phylogenetic tree effectively illustrated the phylogenetic relationships among the 12 insect species. Notably, *B. rhynchopetera* clustered with other coleopteran, including *T. molitor* and *T. castaneum*, findings that are consistent with previous research that employed the mitochondrial genome of *B. rhynchopetera* for phylogenetic tree construction.^11^ Based on the species divergence time, it is demonstrated that the Tenebrionidae lineage diverged from other coleopteran families at about 173.9 Mya. Furthermore, the divergence of the ancestral lineage leading to *B. rhynchopetera* and its closely related species, *T. molitor*, occurred at approximately 85.6 Mya. An evolutionary analysis of gene families revealed that *B. rhynchopetera* exhibited 352 expanded and 611 contracted gene families when compared with *T. molitor* (Fig. 2C).

### 3.5. Synteny

**Fig. 3.**
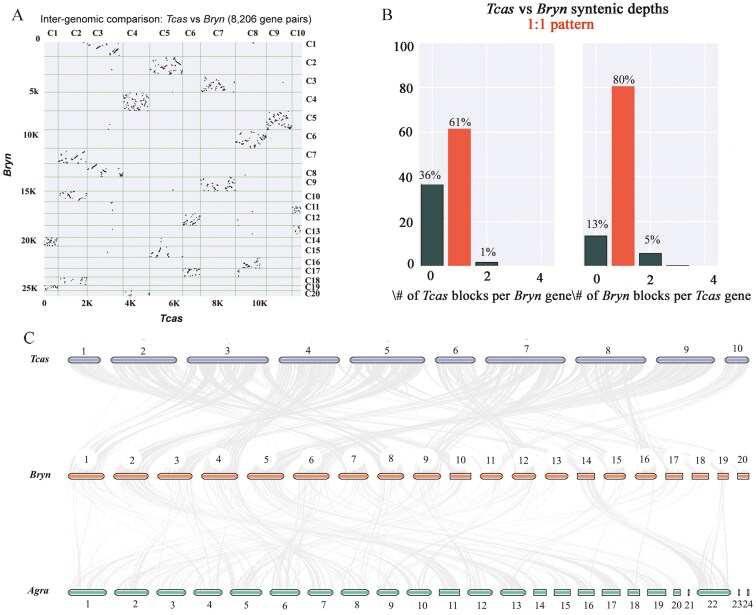
Synteny analysis. A) Scatter plot of *B. rhynchopetera* versus *T. castaneum*. B) Syntenic depth ratio analyses of *B. rhynchopetera* versus *T. castaneum*. C) Synteny analysis of *T. castaneum*, *B. rhynchopetera*, and *A. grandis*.

In order to investigate possible chromosome fusion and fission events, the genomes of *B. rhynchopetera* and the model insect *T. castaneum* were analysed for collinearity with a total syntenic depth ratio of 1:1 (Fig. 3A–C). Obviously, the synteny relationship between chromosome 7 of *T. castaneum* and chromosomes 3 and 9 of *B. rhynchopetera* might indicate one fission event in *B. rhynchopetera*. Similarly, chromosomes 1 and 8, 14 and 19, and 12 and 17 of *B. rhynchopetera* might represent 3 additional fission events. Although various chromosome breakages might have occurred during the evolutionary history, it exhibits a good synteny relationship with *T. castaneum*. In functional genomics research, it is generally believed that collinear genes are more likely to have similar biological functions, and subsequent exploration can be conducted in conjunction with high-quality genomes of *B. rhynchopetera*. We also performed a genome-wide synteny analysis between *B. rhynchopetera* and *A. grandi* (Fig. 3C). Notably, we discovered a poor synteny relationship between these 2 species, possibly resulting from the relatively long phylogenetic distance between them. This also demonstrated the reliability of the phylogenetic tree that we constructed.

### 3.6. GO and KEGG enrichment analysis

The GO and KEGG enrichment analysis conducted on the unique gene families of *B. rhynchopetera* showed that these gene families are mainly associated with DNA recombination, catalytic activity, olfactory receptor activity, odorant binding, and other functions, as indicated by GO enrichment analysis (Supplementary Fig. S3). Furthermore, the analysis also suggested that these gene families are involved in the lysosome, ABC transporters, Toll and Imd signalling pathway, fatty acid metabolism, and other signalling pathways, as suggested by KEGG enrichment analysis (Supplementary Fig. S3). An analysis of the expanded gene families through the KEGG enrichment process has identified their significant enrichment in insect hormone biosynthesis, ABC transporters, terpenoid backbone biosynthesis, thiamine metabolism, arachidonic acid metabolism, and lysine degradation (Table 3). PSGs play a crucial role in the emergence of new functions in species. Utilizing positive selection analysis, we identified 84 PSGs in *B. rhynchopetera*. Subsequently, we conducted GO and KEGG analyses on these genes. The GO enrichment analysis revealed that 84 PSGs were enriched in RNA helicase activity, cell development, and ATP hydrolysis activity (Supplementary Table S8), while the KEGG enrichment analysis indicated that these genes were enriched in mRNA surveillance pathway, inositol phosphate metabolism, and MAPK signalling pathway-fly (Supplementary Table S9). Moreover, expansion and contraction of gene families and PSGs may be associated with adaptive evolution and speciation of *B. rhynchopetera*.

**Table 3.** KEGG enrichment results of *B. rhynchopetera* expanded gene families.

ID	Description	GeneRatio	BgRatio	*P* value	*Q* value	Count
ko00981	Insect hormone biosynthesis	33/164	68/3381	1.12E-26	8.25E-26	33
ko02010	ABC transporters	31/164	88/3381	5.91E-20	2.18E-19	31
ko00900	Terpenoid backbone biosynthesis	25/164	55/3381	2.02E-19	4.95E-19	25
ko00730	Thiamine metabolism	14/164	28/3381	5.13E-12	9.45E-12	14
ko00590	Arachidonic acid metabolism	13/164	31/3381	4.96E-10	6.09E-10	13
ko00310	Lysine degradation	16/164	58/3381	6.16E-09	6.49E-09	16
ko00232	Caffeine metabolism	7/164	10/3381	5.92E-08	5.45E-08	7
ko00230	Purine metabolism	21/164	119/3381	1.35E-07	1.10E-07	21
ko04624	Toll and Imd signalling pathway	18/164	98/3381	6.15E-07	4.53E-07	18
ko00511	Other glycan degradation	11/164	38/3381	9.64E-07	6.46E-07	11
ko00600	Sphingolipid metabolism	11/164	44/3381	4.77E-06	2.93E-06	11
ko00982	Drug metabolism-cytochrome P450	13/164	79/3381	8.23E-05	4.66E-05	13
ko00980	Metabolism of xenobiotics by cytochrome P450	13/164	82/3381	0.000122383	6.44E-05	13
ko00480	Glutathione metabolism	13/164	87/3381	0.000226962	0.00011149	13
ko04146	Peroxisome	11/164	102/3381	0.009569239	0.004406886	11

The unique and expanded gene families were enriched in pathways related to detoxification (ABC transporters, drug metabolism-cytochrome P450, metabolism of xenobiotics by cytochrome P450) and immunity (Toll and Imd signalling pathway), which are beneficial for understanding the environmental adaptation of *B. rhynchopetera*. The cytochrome P450 monooxygenase (P450) and ABC transporters can assist insects in metabolizing xenobiotics, synthetic insecticides, environmental pollutants, and endogenous compounds.^54–56^ Interestingly, the defence glands of *B. rhynchopetera* appear to be closely associated with its detoxification system. For instance, it has been reported that the larvae of *Chrysomela populi* sequester some metabolites through ABC transporters into their defence glands as a successful detoxification strategy.^57^ In addition, Ding et al.^12^ found that differentially expressed genes in the defence glands of *B. rhynchopetera* were significantly enriched in P450-related metabolism, and the downstream products of the ‘Metabolism of xenobiotics by cytochrome P450’ pathway structurally resemble some substances reported in the defensive secretions of *B. rhynchopetera*.^8^ Therefore, we speculate that its defence glands play a significant role in the detoxification system and may also participate in sequestering the metabolites of ABC transporters and P450 from xenobiotics. The Toll and IMD signalling pathways are key components of the innate immune systemic response in insects, capable of responding to fungal and bacterial infections and controlling the expression of antimicrobial peptide (AMP) genes.^58–60^ AMPs are cationic small peptide molecules produced by multicellular organisms as an ancient weapon to effectively prevent infections by bacteria, fungi, viruses, and other pathogens.^61,62^ These detoxification and immunity-related pathways, along with the enriched genes, may facilitate *B. rhynchopetera* in responding to and adapting to the constantly changing environment. It is worth conducting further in-depth research in this area to better understand the mechanisms of environmental adaptation in organisms.

## 4. Conclusion

In this study, we have reported the chromosomal-level genome assembly of *B. rhynchopetera*, an important medicinal insect species. The gene families related to immunity and detoxification are beneficial for understanding its environmental adaptation, which will serve as a valuable research resource for pest control strategies and conservation efforts of beneficial insects. Furthermore, the chromosome-level genome of *B. rhynchopetera* offers fresh perspectives on the genomics of Coleoptera, providing valuable references for comparative genomic studies among insects. In addition, it will support more reliable genomic-level investigations into the behavioural patterns of *B. rhynchopetera*, assisting farmers and traders in devising breeding strategies.

## Supplementary Material

dsae027_suppl_Supplementary_Figures

dsae027_suppl_Supplementary_Tables

## Data Availability

The raw data and the chromosome-level genome assembly of *B. rhynchopetera* have been deposited to the National Center for Biotechnology Information (NCBI, https://www.ncbi.nlm.nih.gov),^10^ with the BioProject accession PRJNA1040117 and the BioSample accession SAMN38241943. In addition, the genome assembly is available under accession number JAYJMQ000000000.
