# Correction to: Chromosome-level genome assembly of the medicinal insect *Blaps rhynchopetera* using Nanopore and Hi-C technologies

**DOI:** 10.1093/dnares/dsae037

**Published:** 2024-12-27

This is a correction to: Wei Zhang, Yue Li, Qi Wang, Qun Yu, Yuchen Ma, Lei Huang, Chenggui Zhang, Zizhong Yang, Jiapeng Wang, Huai Xiao, Chromosome-level genome assembly of the medicinal insect *Blaps rhynchopetera* using Nanopore and Hi-C technologies, *DNA Research*, Volume 31, Issue 6, December 2024, dsae027, https://doi.org/10.1093/dnares/dsae027

The originally published version listed all authors as being affiliated with the School of Artificial Intelligence and Information Technology, Nanjing University of Chinese Medicine, Nanjing 210023, Jiangsu Province, People’s Republic of China. Only the second author is affiliated with this institution. The article has been updated to reflect this.

