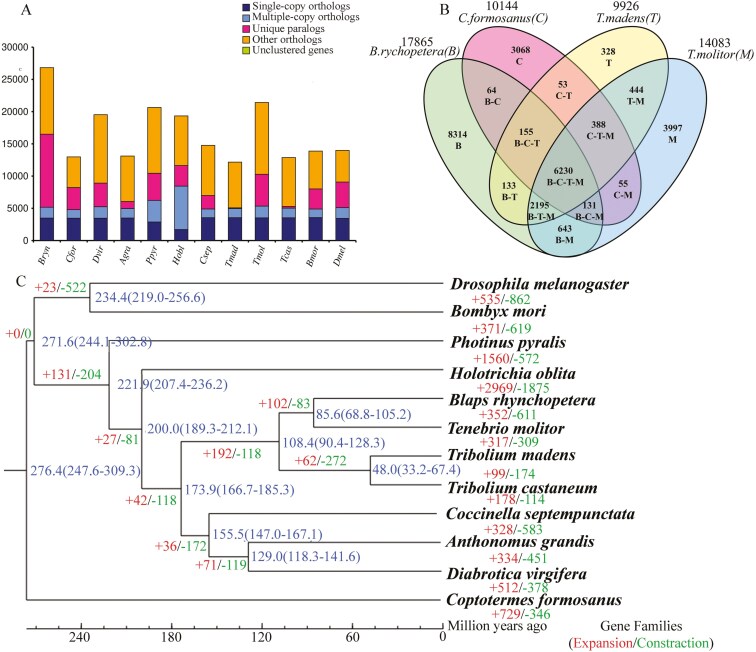# Correction to: Chromosome-level genome assembly of the medicinal insect *Blaps rhynchopetera* using Nanopore and Hi-C technologies

**DOI:** 10.1093/dnares/dsaf013

**Published:** 2025-06-04

This is a correction to: Wei Zhang, Yue Li, Qi Wang, Qun Yu, Yuchen Ma, Lei Huang, Chenggui Zhang, Zizhong Yang, Jiapeng Wang, Huai Xiao, Chromosome-level genome assembly of the medicinal insect *Blaps rhynchopetera* using Nanopore and Hi-C technologies, *DNA Research*, Volume 31, Issue 6, December 2024, dsae027, https://doi.org/10.1093/dnares/dsae027

During preparation of figure 2, the species *Tenebrio molitor* was inadvertently listed twice. The second instance of this name has been corrected to read *Tribolium madens*.