# Supplementary material for: Chromosome-level genome assembly of the medicinal insect Blaps rhynchopetera using Nanopore and Hi-C technologies
Source: DNA Res. 2024 Sep 9;31(6):dsae027. doi: 10.1093/dnares/dsae027 (PMC11555684; doi:10.1093/dnares/dsae027)
Supplement: dsae027_suppl_Supplementary_Figures [file dsae027_suppl_supplementary_figures.docx]

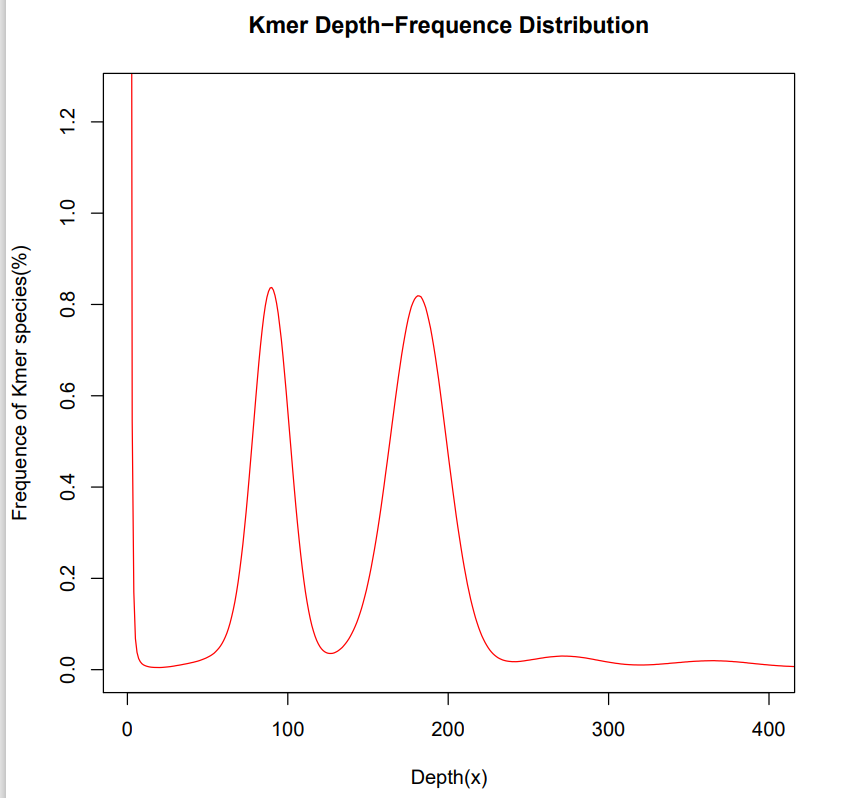


**Figure S1.** K-mer=19 distribution curve. The horizontal coordinate indicates the depth with Kmer = 19, and the vertical coordinate is the frequency of different depths.


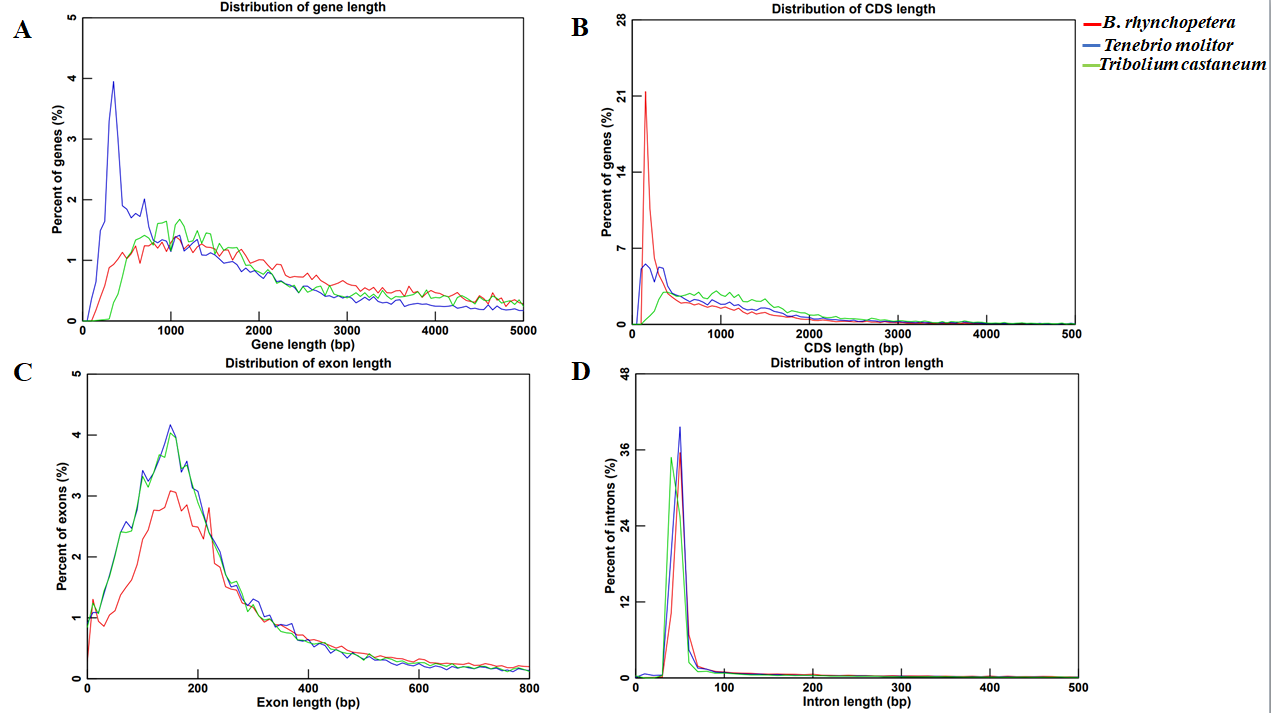


**Figure S2** Comparison of gene lengths,CDS lengths, Exon lengths, Intron lengths among *B. rhynchopetera* and close species. The horizontal coordinates in the four graphs represent gene lengths, CDS lengths, intron lengths, and exon lengths (win=10bp), respectively. The vertical coordinate represents the number of genes of statistical length as a percentage of the total number of genes.


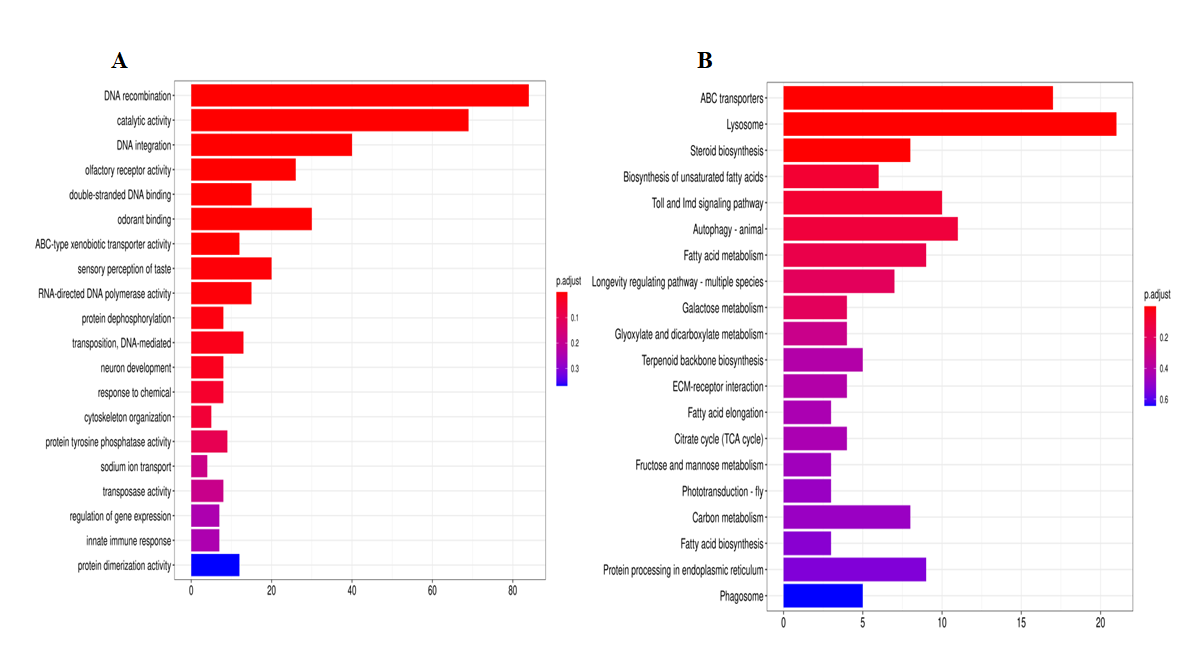


**Figure S3** Enrichment analysis of the *B. rhynchopetera* unique gene families. (**A)** GO enrichment results of unique gene families: Top 20 GO categories were shown; **B**. KEGG enrichment results of unique gene families: Top 20 GO categories were shown. In these two diagrams, the vertical axis indicates the enrichment pathway and the horizontal axis indicates the number of genes; the colors represent different *P* values.
